# Evaluation of Groundwater Recharge Estimates in a Partially Metamorphosed Sedimentary Basin in a Tropical Environment: Application of Natural Tracers

**DOI:** 10.1155/2014/419508

**Published:** 2014-03-19

**Authors:** Felix Oteng Mensah, Clement Alo, Sandow Mark Yidana

**Affiliations:** ^1^Department of Earth and Environmental Studies, Montclair State University, Montclair, NJ 07043, USA; ^2^Department of Earth Science, University of Ghana, Legon, Accra, Ghana

## Abstract

This study tests the representativeness of groundwater recharge estimates through the chloride mass balance (CMB) method in a tropical environment. The representativeness of recharge estimates using this methodology is tested using evaporation estimates from isotope data, the general spatial distribution of the potential field, and the topographical variations in the area. This study suggests that annual groundwater recharge rates in the area ranges between 0.9% and 21% of annual precipitation. These estimates are consistent with evaporation rates computed from stable isotope data of groundwater and surface water in the Voltaian Basin. Moreover, estimates of groundwater recharge through numerical model calibration in other parts of the terrain appear to be consistent with the current data in this study. A spatial distribution of groundwater recharge in the area based on the estimated data takes a pattern akin to the spatial pattern of distribution of the hydraulic head, the local topography, and geology of the terrain. This suggests that the estimates at least qualitatively predicts the local recharge and discharge locations in the terrain.

## 1. Introduction 

In regional hydrogeological studies and groundwater resources assessments, accurate estimates of groundwater recharge are required to ensure proper water balance studies and evaluation of groundwater resources for productive uses. Recharge is one of the uncertain parameters in model calibration and is regarded as one of the major parameters which determine the accuracy and reliability of predictive groundwater flow models. It is therefore apposite that this component of basin wide regional hydrogeological investigations is constrained through a variety of methods. Several methods have been proposed in the literature for providing reliable estimates of groundwater recharge at both the regional and local scales. They range from direct measurements through mass balance techniques and Darcian method, to the use of tracers [1, 2]. A wide variety of mass balance techniques are available and their utilities are often based on an assessment of the validities of underlying assumptions in the areas where such applications are sought. Rorabaugh and Meyboom [3] developed methodologies that are based on baseflow recession and assume amongst others that the baseflow component of stream flow can be approximated to groundwater recharge in large basins, especially where such a hydraulic connection can be established between stream flow and the underlying aquifers. Using this methodology, there is a high possibility of overestimating groundwater recharge rates especially where the catchment area of the stream is larger than the domain being examined. The water table fluctuations method [4] has also been advanced and used in several areas to estimate groundwater recharge in unconfined aquifers. The method is based on the assumption that appreciation in groundwater levels between seasons is attributed to groundwater recharge, which is proportional to the specific yield of the material. Therefore, where the aquifer is confined or semiconfined, the methodology is less appropriate and will lead to underestimation or overestimation depending on the values of the specific yield used. Even where the aquifer is unconfined, the spatial variability in the specific yield field can lead to significant departures of recharge estimates from the reality on the ground. The method is often used in places where initial estimates are required for much more detailed regional hydrogeological studies. Several tracer techniques have been proposed in the literature and they range from the application of isotope techniques to the use of natural hydrochemical conservative tracers such as the chloride ion [2]. The use of tracers to explain groundwater recharge and flow processes is copiously documented in the literature [5]. The chloride mass balance (CMB) technique is based on the assumption that the chloride ion behaves conservatively and is not easily affected by reactions through the unsaturated zone through to the saturated zone. If this assumption is valid, then it follows that the ion can adequately trace groundwater recharge processes and can thus provide reasonable estimates of groundwater recharge in the area. Its reliability therefore hinges on the compatibility of the precipitation events that recharged the system under study and recent precipitation. It is also assumed that the main source of chloride in groundwater is precipitation. Therefore, where it can be determined that a substantial proportion of the groundwater chloride is generated from mineral dissolution processes, the method can lead to underestimation of recharge. On the other hand, where the groundwater chloride is negatively affected by a process which reduces its content compared to that of precipitation, groundwater recharge can potentially be underestimated. The CMB methodology has been widely tested and regarded as one of the most reliable techniques for estimating groundwater recharge in regional hydrogeological studies and basin wide groundwater resources assessments (e.g., [5–10]).

The current study evaluates the performance of the CMB methodology in a typical tropical climatic environment where the availability of groundwater resources is critical to socioeconomic conditions of populations and the survival of ecosystems that depend on such groundwater resources for sustenance. Groundwater recharge estimates from the CMB method in this study are checked against estimates of evaporation rates of percolating rainwater as estimated from isotope techniques. The spatial pattern of variation in the resulting recharge estimates is then predicted using a spatial prediction method for the resource to be evaluated. The novelty lies in the fact that the CMB method, although not new, is rigorously evaluated using another conservative tracer.

## 2. The Study Area

**Figure 1:**
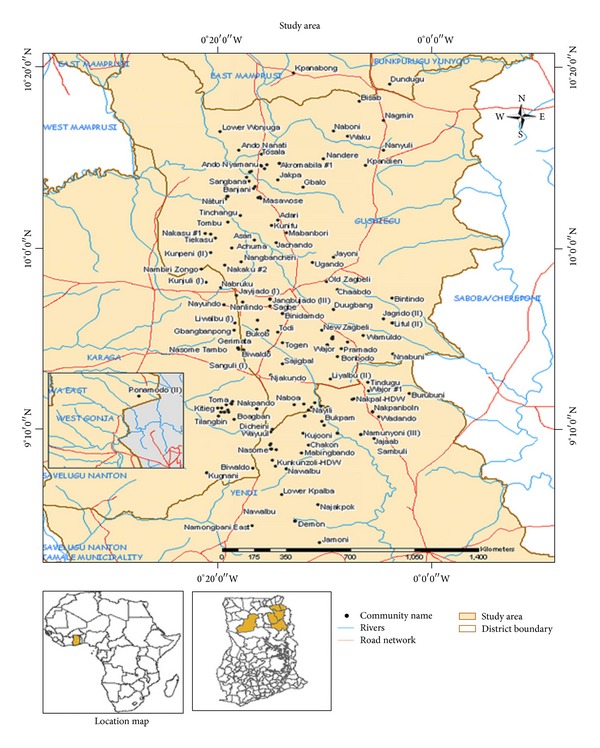
A map of the study area showing the major communities and settlements.

The study area (Figure 1) is in the Northern Region of Ghana and covers a total land area of about 1790.7 Km^2^, and it falls within the White Volta Basin. The agricultural sector is the largest employer in the area, employing about 97% of the active populations [11]. Most of the farmers depend on rain for food production and the typical crops grown include yam, maize, rice, groundnut, cowpea, and soya beans. Unfortunately, agricultural activities are constrained by unreliable rainfall, inadequate irrigation facilities, and difficulty in loan accessibility and lack of storage or processing units leading to postharvest losses [12]. The area is generally flat with gently undulating reliefs. There are no high mountains in the area except few hilly features observed in the southern part with elevations generally ranging between 122 and 244 m above sea level; however, the north is low lying. The area is mainly drained by the White Volta and its tributaries. It falls within the Sudan Savannah zone with an annual rainfall between 900 and 1,200 mm distributed on average over 74 rainy days [13]. The study area falls under the Intertropical Convergent Zone, the interface where two air masses, tropical continental and tropical maritime, overlap. The frontal activity and relative movement of the two air masses control the amount and duration of rainfall. Rainfall, which is generally of short duration and high intensity and is often preceded by thunderstorms and line squalls starts intermittently between March and April through August to September when it turns stable and very heavy. The dry seasons are pronounced with temperature ranging between 18 and 42°C with mean value of approximately 30°C [14]. Relative humidity during wet/rainy season is in the range 40 and 70% and drops to about 15% during the rest of the year [13]. The observed low humidities and high temperatures have led to high potential and actual evapotranspiration rates in the basin.

**Figure 2:**
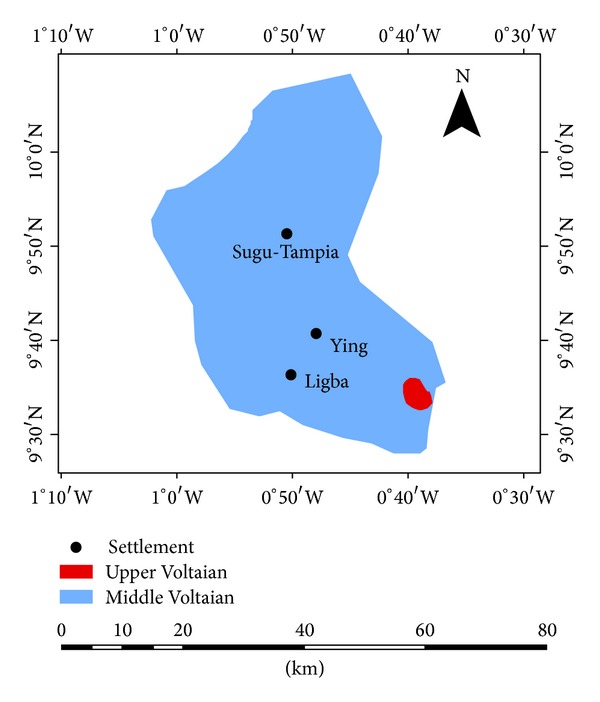
A geological map of the study area.

**Figure 3:**
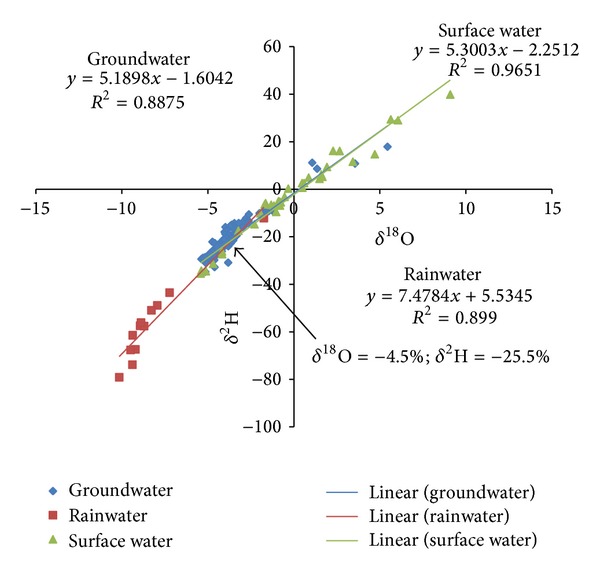
Stable isotope signatures of rainwater, surface water, and groundwater from parts of the White Volta Basin, Ghana.

The area is underlain by consolidated Neoproterozoic sedimentary sequences of the Voltaian Super-Group (Figure 2). The rocks are mainly of the Middle Voltaian (Figure 3), which is the most extensive sedimentary formation in Ghana. The Middle Voltaian comprises the Oti and the Obosum beds, which are well consolidated and generally flat lying. The beds are made up of interbedded mudstones/siltstones, sandstones, arkoses, and conglomerates [15]. Details of geology and hydrogeology are documented in detailed reports and articles on the basin [14, 16–21]. The rocks are largely impervious, so that the occurrence of groundwater is associated with the development of secondary porosity through jointing, shearing and fracturing, and weathering. Rock compaction and slight metamorphism is believed to have destroyed the primary porosity [16], leading to considerably reduced inherent primary permeabilities. However, where intense weathering occurs, the rocks serve as better aquifers with significantly enhanced hydraulic and storage properties. The nature, aperture, and degree of interconnection between joints determine the hydrogeological fortunes of the rocks [14, 17–19]. Drilling projects and hydrological investigations reveal that shallow potential aquifers capable of delivering water of sustainable quantities for domestic and industrial use exist in the basin [20].

Peasant rain-fed agriculture is the main source of employment in the basin. However, recent erratic patterns of rainfall in the area, coupled with rising populations in the basin, have led to increased interests in developing the irrigation potentials in the area. This has heightened interests in assessing the potentials of developing the aquifers in the area for commercial abstraction to support irrigation activities to enhance food security and reduce poverty since agriculture is the mainstay of the communities there.

## 3. Materials and Methods

A total of forty-two samples (19 groundwater, 11 rainwater, and 12 surface water samples) were collected for the study. Rainwater was taken in Tamale and analyzed for both the chloride and isotopes (^18^O and ^2^H). Historical groundwater hydrochemical data from the basin were obtained from the Community Water and Sanitation Agency (CWSA) in the Northern Region. The surface water samples were taken from tributaries of the White Volta. Strict adherence to sampling protocols was followed for collected water samples both in the field and the laboratory. All the analyses were carried out at the Ghana Atomic Energy Commission's chemistry laboratory. Chloride analysis was done using a Dionex ICS 90 ion chromatograph equipped with an AS14A-5 µm ion pac column. Stable isotope analysis of *δ* ^18^O was carried out using VG Sira 10 mass spectrometry. Delta deuterium measurements were done on a EuroVector elemental analyzer (EA; EuroPyrOH-3100) with a liquid autosampler (LAS; Euro AS-300) coupled to a Micromass IsoPrime isotope ratio mass spectrometer. The isotope composition of water is reported as the deviation of ^2^H/^1^H or ^18^O/^16^O ratio from that of Vienna Standard Mean Ocean Water (VSMOW) in parts per thousand (‰).

The CMB methodology is summarized in

(1) $$\begin{array}{c} \text{Recharge}=\frac{C_{p}}{C_{\text{gw}}}P, \end{array}$$

where  *C*_*p*_  and  *C*_gw_,  respectively, represent chloride concentrations in precipitation and groundwater and *P* represents the average annual depth of precipitation in the area.

Average chloride concentration in rainwater was obtained from analyses of rainwater samples taken in Tamale, the regional capital of the Northern Region, as part of this study. Groundwater chloride concentration was obtained from the historical hydrochemical data that had already been procured from the CWSA. An average annual precipitation rate of 1100 mm was used in the estimation. Ordinary Kriging was then applied to the estimated recharge to achieve a regional distribution in the area. Ordinary kriging is a linear estimation method which is based on [22]

(2) $$\begin{array}{c} Z\left(x\right)=\overset{n}{\underset{i=1}{\sum }}\lambda_{i}Z\left(x_{i}\right), \end{array}$$

where *λ*_*i*_ refers to the weight assigned to a known or estimated value *Z*(*x*_*i*_), of the parameter, and *Z*(*x*) is the new value being estimated.

Different estimation methods use different criteria to assign the weights to parameters, but most of them are based on the proximities of the known locations to the locations being estimated [22]. Where proximity is the sole determinant, the weights are assigned such that closer points have higher contributions to the estimates than farther points as is the case with inverse distance weighting [22]. The advantage of ordinary kriging over the others is that it uses both the proximity factor and the general pattern of spatial variation of the parameter with distance. A variogram is modeled from the original data as part of the processes of estimating the weighting factors or coefficients. Such a model characterizes the spatial dependence of the parameter in the domain. It is the fitted experimental model which is then used together with the proximity factor to estimate coefficients for all the data to be used in the estimation.

In this study, both the variography and ordinary kriging were performed from the R-platform. An acceptable variogram was carefully chosen and fitted to the computed dataset of the recharge estimates. This was achieved by trying the various possible theoretical models available until the most appropriate model was achieved. Ordinary kriging was then performed from the variogram model chosen. Prediction accuracy using ordinary kriging and any kriging methodology partly hinges on how adequately the spatial dependence of the parameter was modeled. This implies that the choice of an inappropriate model of spatial dependence can potentially lead to inaccurate estimates or predictions.

## 4. Results and Discussions

The sources and origin of groundwater recharge in the Voltaian was assessed using stable isotope data of precipitation (rainfall), groundwater, and surface water from parts of the Voltaian. Historical stable isotope data (*δ* ^18^O and *δ* ^2^H) for groundwater, surface water, and rainfall were plotted on a biplot for the purposes of ascertaining the sources and/or evolution of groundwater in the area. Isotope tracers are amongst the most frequently used to trace the sources and/or genesis of water reservoirs and contaminants and have been noted to provide useful insights to guide further detailed investigations. The ratio of the rarer (and most often the heavier) isotope to the more abundant (often the lightest) isotope provides an indication of the relative enrichment of the two isotopes in the medium or the original source of recharge. It is such a ratio that provides indications of the climatic conditions prevailing at the period and location of recharge and can therefore be used qualitatively to infer the age of a water body. These ratios are expressed in relation to an international standard which provides some uniformity for comparing environments or reservoirs on a global scale and is often expressed in the delta (*δ*) notation as indicated in

(3) δO18=((O18/O16)sample(O18/O16)V-SMOW)×1000,δH2=((H2/H1)sample(H2/H1)V-SMOW)×1000,

where the terms in the numerator and denominator, respectively, represent the ratio of the heavier to the lighter isotope in the sample and international standard, respectively.

The global meteoric water line (GMWL) was first published by Craig [23] and is a convenient reference for understanding and tracing water origin. It is a linear relation in the form of

(4) $$\begin{array}{c} \delta^{2}\text{H}=8\delta^{18}\text{O}+d, \end{array}$$

where *d*, the *y*-intercept, is the deuterium excess (or *d*-excess) parameter when the slope = 8 [24]. From Craig's MWL, *d* = 10 at this slope and is indicative of no evaporative effect during precipitation. The underlying assumption [23] is that water with an isotopic composition that falls along the GMWL originates from the atmosphere and is relatively unaffected by isotopic processes. Isotopic signatures from different reservoirs are often discussed in relation to (4). In this study, groundwater recharge in the area appears to be of meteoric origin as the groundwater data plots close to the GMWL, albeit with shallower slope and intercept, which suggest some degree of enrichment of the heavier isotope relative to the lighter ones (Figure 3). The observed pattern is consistent with conditions of lower relative humidity than 100% and high ambient temperatures as is common in the study area. The shallower slope and *d*-excess values result from evaporation of raindrops due to low relative humidities during the course of the rains. The average relative humidity in the area during the rainy season is about 70% [13]. Vertical percolation of precipitation down to the saturated zone is variable in the space of the study area, due to the variability in the clay content which limits the vertical hydraulic conductivity of the material of the unsaturated zone. Where the clay content is high, vertical percolation is significantly restricted, and the resulting recharged groundwater is significantly enriched due to evaporative effects of high temperatures and low humidities. It is obvious that the surface water data fall exactly on the Global Meteoric Water Line, GMWL, whereas both the rainwater and groundwater samples are relatively enriched, with shallower slopes and deuterium excess (*d*-excess) values (Figure 3). The rainwater samples were taken during three events in the major rainy season and may not adequately reflect the signature for the entire year. However, studies conducted in the southern parts of the basin using data mainly of the dry season precipitation suggest significant enrichment [25]. A solution of the equation for the Local Meteoric Water Line (LMWL) developed from the precipitation data and the local Groundwater Line (LGWL) developed from the groundwater isotope data provides the isotopic signature of the most probable source precipitation of the groundwater in the area. The intersection of the LGWL and LMWL in Figure 3 provides such a signature. As is obviously presented in Figure 3, the isotopic signature at the point of intersection of these two lines (*δ* ^18^O = −4.5; *δ* ^2^H = −25.5) suggests that the source of recharge is recent meteoric water or a mixture of precipitation types which are of recent origin. The local surface water line (LSWL) is similar to the groundwater line in terms of slope and intercept (Deuterium excess) and suggests that both have a similar source and may have been affected by similar processes over time. A recent assessment of the isotope characteristics of the entire Voltaian indicated a similar isotopic signature of the source water of groundwater recharge in the basin [26] and indicated that the processes of infiltration and percolation of precipitation water may be variably slow in the terrain, due to the variability in the nature of the overburden even within short distances. Yidana [26] estimated the rates of evaporation of precipitation water in transit down the unsaturated zone to the saturated zone and reports evaporative losses of 19.8%–70.6% for the Voltaian sedimentary basin in Ghana. This contrasts with significantly higher evaporation rates estimated for surface water bodies in the area (29.5%–84.7%) and highlights the fact that surface water, in view of its continuous exposure to the atmosphere endures higher impacts of higher ambient temperatures and low humidities than infiltrating water. In both cases, however, the estimates are most likely the average conditions over a long period of time since the waters sampled are most likely mixed waters from different events. These estimates do not include the effects of transpiration as the impacts of transpiration on isotopic signature of water are difficult to estimate. However, transpiration contributes significantly to water loss especially in the unsaturated zone. Therefore, if included, the rate of water loss due to the combined effects of evaporation and precipitation in the entire terrain and in the study area will be significantly higher.

Estimated groundwater recharge from the CMB suggests annual recharge in the range of 0.9%–21% of the total annual precipitation. This is consistent with the observation of high evaporation rates estimated for the entire basin and suggests that much more water may have been lost to transpiration. However, this much of groundwater recharge suggests high fortunes in terms of commercial groundwater resources development in the area, if a significant proportion of it is available for abstraction. The wide range in the estimated data and the high standard deviation suggests significant spatial variation in groundwater recharge rates in the study area. This may be related to the nature of the unsaturated zone material and its variability in the space of the domain of this study. The average rate of about 5.5% is significant and compares favorably estimates in other parts of the terrain. For instance Attandoh et al. [11] estimated groundwater recharge in the range of 0.3%–4.1% of total annual precipitation in the area, through model calibration in the same terrain. Earlier estimates of Yidana [27] in the south of the Voltaian suggested similar rates through model calibration.

**Figure 4:**
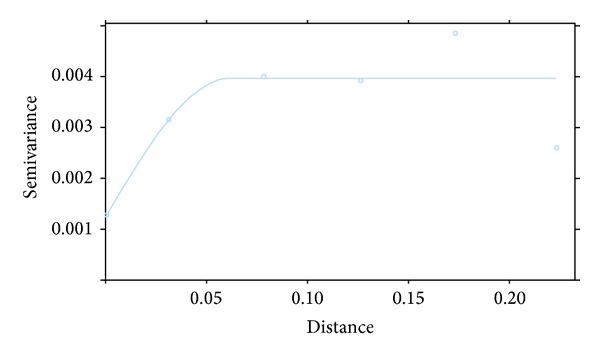
Semivariogram for estimated groundwater recharge in the study area.

**Figure 5:**
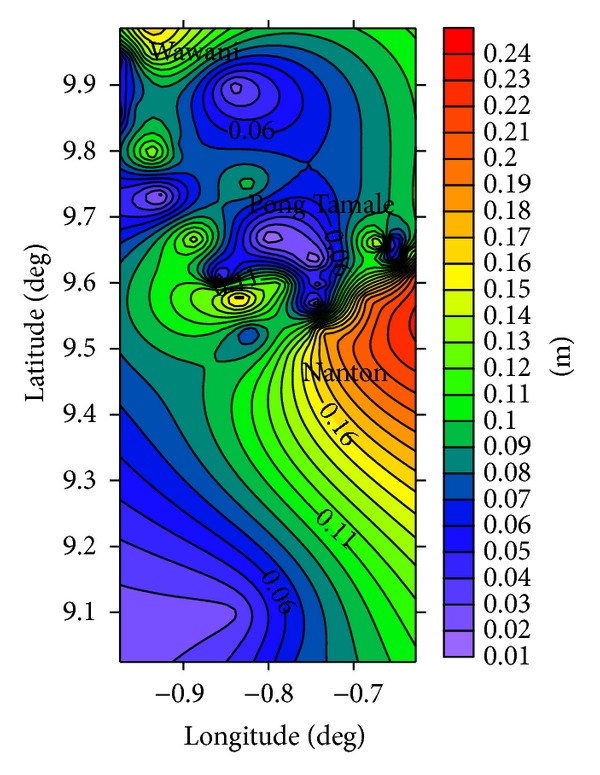
Spatial distribution of estimated groundwater recharge in Savelugu and surrounding subcatchments of the White Volta Basin.

Ordinary kriging was employed to predict the spatial pattern of variation in groundwater recharge in the study area.  Figure 4 presents the variogram that was fitted to the original, estimated groundwater recharge rates from the CMB methodology. A spherical variogram was adjudged appropriate for the distribution and was accordingly chosen for the estimation stage of ordinary kriging. This ensured that the prediction is as accurate as possible. The resulting predicted surface for the spatial variation in recharge in the domain is presented in Figure 5. A significant spatial pattern of variability in groundwater recharge is suggested in Figure 5.

There is an apparently high variability in direct groundwater recharge from precipitation in the area and much of the Voltaian basin. This observation is consistent with the nature of the material in the unsaturated zone which varies in space in terms of the clay content. Where the clay content is considerably high, vertical percolation of rainwater is much reduced, leading to reduced vertical recharge. Infiltrating rainwater experiencing such restricted vertical flow therefore undergoes significant evaporation such that a high percentage is lost to the atmosphere. Yidana [26] suggests that, on the basis of the analyses of stable isotope data of precipitation, rainwater, and groundwater from parts of the entire basin, evaporation of infiltrating rainwater is in the range of 19.8% and 70.6% of the total annual precipitation in the basin. This is quite consistent with the observed low humidities and high annual temperatures and explains why surface impoundments have not been successful as sustainable sources of irrigation water in the region.

**Figure 6:**
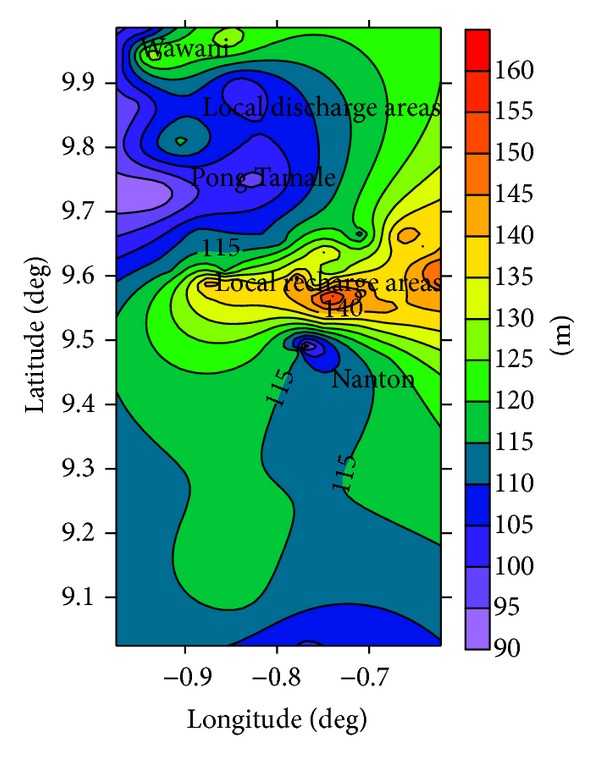
Potentiometric map of the study area showing local recharge and discharge areas.

**Figure 7:**
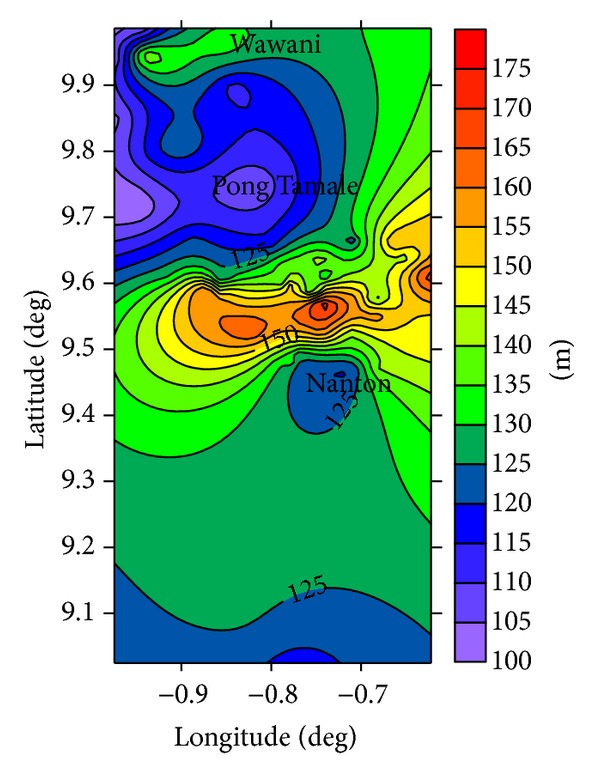
Local topographical map of the study area.

The estimated groundwater recharge distribution appears to be consistent with configuration of the groundwater flow pattern suggested by the groundwater table map (Figure 6) and the surface topographical map (Figure 7) of the area. The highest recharge areas (Figure 5) are generally in the eastern parts of the area, which coincide with the local recharge areas suggested by the potentiometric map (Figure 5).

Some of the areas of low recharge in the north and south of the study area (Figure 5) also coincide with locations of the lowest hydraulic head (Figure 6) and suggests that the groundwater recharge rates from the CMB methodology accurately accounts for the observed groundwater flow geometry in the area. Groundwater recharge estimates from the CMB methodology is largely accurate when applied in arid to semiarid areas where local surface runoff is negligible [28]. Yidana and Koffie [29] applied the same methodology to similar aquifers in the north of the area and suggested groundwater recharge in the range of 1.8% and 32% of the total annual rainfall in the area. These variable recharge rates predicted in their study are consistent with the nature of the material in the unsaturated zone as suggested by some well logs in the area.

Estimating one of the key hydrogeological parameters is based on a proper understanding of estimates of other uncertain parameters in the terrain in hydrogeological modeling. In the study of Attandoh et al. [11], the key parameters of aquifer hydraulic conductivity and recharge were estimated through model calibration. Previous researchers [24, 30] criticized the estimation of key parameters of groundwater flow in this pattern, contending that a wide range of values could produce the same level of calibration, leading to the lack of uniqueness in such estimates. The range of estimates obtained from the current study provides a wider range and probably accounts for the marked variability in the nature of the material in the unsaturated zone, which regulates groundwater recharge. In addition, as suggested in the study of Attandoh et al. [11] much of the recharge was computed through the general head boundary. In effect, when all the components of recharge estimated from the model are put together, the range is compatible with those computed with the CMB methodology in this study. This suggests that, in spite of the criticisms of nonuniqueness in parameter estimates through model calibration, the methodology provides quite reliable estimates if carefully executed.

## 5. Conclusion 

Results of this study suggest that the chloride mass balance (CMB) approach performs well in a tropical setting in providing fairly accurate estimates of groundwater recharge for groundwater resources evaluation. Estimates in this study compare well with estimates from numerical model calibration and analyses of evaporation rates of infiltrating rainwater. Estimated groundwater recharge from the CMB indicates that 0.9% to over 21% of annual precipitation recharges the shallow aquifer system in the area. This much of groundwater recharge suggests high fortunes in terms of commercial groundwater resources development in the area. The study also indicates that the source of groundwater recharge in the terrain is recent meteoric water which may have been isotopically enriched in the heavier isotopes of the elements of the water molecule during the process of recharge. It is suggested in this study that, whenever the CMB methodology must be used in groundwater recharge estimation, other tracers must be used to check the validity of the results as indicated in this study.
